# Health characteristics associated with chemsex among men who have sex with men: Results from a cross-sectional clinic survey in Norway

**DOI:** 10.1371/journal.pone.0275618

**Published:** 2022-10-05

**Authors:** Eirik Amundsen, Åse Haugstvedt, Vegard Skogen, Rigmor C. Berg

**Affiliations:** 1 Department of Clinical Medicine, Faculty of Health Sciences, UiT The Arctic University of Norway, Tromsø, Norway; 2 The Olafia Clinic and the National Advisory Unit on Sexually Transmitted Infections, Oslo University Hospital, Oslo, Norway; 3 Department of Infectious Diseases, Division of Internal Medicine, University Hospital of North Norway, Tromsø, Norway; 4 Department of Community Medicine, Faculty of Health Sciences, UiT The Arctic University of Norway, Tromsø, Norway; 5 Division for Health Services, Norwegian Institute of Public Health (NIPH), Oslo, Norway; Washington University in Saint Louis, UNITED STATES

## Abstract

**Background:**

Chemsex typically involves drugs such as GHB/GBL, crystal meth and mephedrone, and is increasingly common among MSM. The behaviour has been found to be associated with sexually transmitted infections (STIs) and mental health problems. We aimed to assess the extent of chemsex engagement and associations with different aspects of health, among MSM attending a free specialist walk-in clinic for STIs in Oslo, Norway.

**Methods:**

Anonymous cross-sectional survey data was collected from June to October 2016. Differences in STI health (chlamydia, gonorrhoea, syphilis, HIV diagnoses), mental health (depression/anxiety) and internalised homonegativity between MSM using and not using GHB/GBL, crystal meth, mephedrone, cocaine or ketamine with sex in the last year were assessed descriptively and in a multivariate logistic regression model. The predictors were number of self-reported chlamydia, gonorrhoea or syphilis diagnoses, HIV diagnosis, depression/anxiety, and degree of internalised homonegativity. We adjusted for age, education level and having lived abroad.

**Results:**

Of the 518 MSM respondents, 17% reported sexualised use of either GHB/GBL, crystal meth, mephedrone, cocaine or ketamine in the last year (chemsex). We found significant positive associations between chemsex and self-reported HIV diagnoses (adjusted odds ratio [aOR] = 3.26, 95%CI = 1.37–7.76), number of reported chlamydia, gonorrhoea or syphilis diagnoses in the last year (aOR = 1.63, 95%CI = 1.18–2.12), having lived more than one year abroad (aOR = 2.10, 95%CI = 1.20–3.65), but no significant association with depression/anxiety (aOR = 1.02, 95%CI = 0.53–1.93), nor internalised homonegativity (aOR = 0.62, 95%CI = 0.33–1.19).

**Conclusion:**

Chemsex engagement in Norway is relatively low compared to findings from STI clinics in other European countries, and GHB/GBL and cocaine the two most commonly used drugs with sex. Chemsex was more common among MSM having lived more than one year abroad, reporting HIV diagnoses and a higher number of either chlamydia, gonorrhoea or syphilis diagnoses in the last year. Health care providers need to be made aware of chemsex as a behavioural phenomenon among MSM, and special care should be afforded to MSM living with HIV and being diagnosed with STIs.

## Introduction

Chemsex refers to recreational drug use in a context that involves sex [1–6], and appears to be more frequent among MSM in comparison to the non-MSM population [1, 6]. The drugs associated with chemsex can be taken to prolong sexual motivation, pleasure and activity, to increase sexual self-confidence, as well as to enhance the perceived quality of sex in other ways [5, 7–11]. Over the last two decades, an increasing number of studies have explored the possible relevance of chemsex to MSM health [12–15].

Although no uniform definition of chemsex exists [12, 16], the norm in the literature is to conceptualise chemsex as the use of specific drugs during sex, including stimulants (crystal meth, mephedrone, cocaine, ecstasy/MDMA, amphetamine), GHB/GBL and/or ketamine. These drugs have different effects, and MSM have reported using them for sex with different intentions, as well as experiencing different health consequences [9, 16–22], depending on the drug or combination of drugs used for sex. Individual MSM chemsex studies have adopted very different operationalisations of chemsex that include some, but not all the chemsex drugs listed above [23, 24]. This reduces comparability between chemsex studies somewhat, but provides an opportunity to explore health differences between MSM who engage in different types of chemsex.

In a recent European internet survey among 128 000 MSM recruited from social and sexual media, which excluded GHB/GBL from its definition of chemsex, 7% of Norwegian MSM reported use of at least one stimulant (crystal meth, mephedrone, cocaine, ecstasy/MDMA, amphetamine) or ketamine with sex in the last year, and 3% in the last month [25]. The equivalent rates for the whole of Europe were 10% and 5% [26], and for Denmark, another Nordic country, 9% and 4% [27]. Increases in chemsex have been seen among both MSM living with [28] and not living with [29] HIV.

A variety of reasons has been proposed as to why chemsex has become a behavioural phenomenon among MSM. MSM reporting recent chemsex engagement are more likely to report recent diagnoses of sexually transmitted infections (STIs), such as chlamydia, gonorrhoea and/or syphilis [7, 14, 30–35] and HIV [33–37]–collectively referred to as STI health in this text–and sexual behaviours that are traditionally presumed to increase the likelihood of STI transmission [1, 3, 38, 39]. Some have found chemsex engagement among MSM to be associated with mental health [7, 30, 38], independently of the relatively well-established association between chemsex and STI health, and others similar findings in bivariate regressions or descriptive analyses [3, 6, 32, 33, 40, 41].

Regarding the underlying health drivers of chemsex, it has been argued that MSM who engage in chemsex do so to compensate for accumulated harm caused by exposures to societal homonegativity–manifesting itself in MSM as a reduced ability to enjoy sex, love and relationships–the physical, mental and social traumas of the HIV/AIDS epidemic, as well as a general intensification of a unique rejection culture among MSM [36]. Syndemic theory and the minority stress model are often cited in MSM chemsex literature, as theoretical frameworks that could explain the possible connection between experiences of societal homonegativity, mental health and chemsex engagement among MSM, as compared with the general population [42–46]. Syndemic theory suggests that co-occurring health inequalities experienced by stigmatised minority groups may be caused by social stigma [39], and the minority stress model similarly highlights the role of ongoing stress and perceived and enacted stigma because of being part of a minority more generally [47, 48]. Non-heterosexual individuals generally, and some groups more than others, experience worse mental health than the general population, reporting for instance higher rates of depression, anxiety, post-traumatic stress, self-harm and substance use disorders [49]. To the best of our knowledge, no studies have examined whether independent associations between chemsex engagement, STI health, mental health and harm from accumulated experiences of societal homonegativity exist among MSM. Additionally, research on chemsex from the Nordic region is scarce. We therefore aimed to examine the extent and characteristics of chemsex engagement, and to explore associations between variations in chemsex engagement, chlamydia/gonorrhoea/syphilis diagnoses, HIV diagnosis, depression/anxiety, and one measure of internalised homonegativity, among MSM attending a walk-in STI clinic in Oslo, Norway.

## Methods

### Population

The present study includes a subset of a larger survey study of MSM and men who have sex with women [50, 51]. The objective of the original study was to assess occurrence and characteristics of chemsex among male STI clinic attendees and variables associated with chemsex. Eligibility criteria for survey participation were: 1) attending the free Oslo University hospital walk-in clinic for testing and treating STIs (the Olafia clinic), 2) at least 16 years of age, 3) male gender, 4) had sex in the last 12 months, and 5) ability to understand written Norwegian or English. For the current analysis, our study population included clinic attendees who met these five eligibility criteria, and in addition self-reported (in the survey) that they had sex with men in the past year.

### Recruitment and procedures

We recruited participants from a free university hospital walk-in STI clinic in Oslo, the capital of Norway, in the summer and fall of 2016. The Olafia clinic is the only STI specialist health service clinic in Oslo. It offers free asymptomatic testing and consultations with physicians in the case of symptoms that may require treatment before laboratory test results are available. Treatment of chlamydia, gonorrhoea, syphilis and HIV are free in Norway.

The Regional Committee for Medical and Health Research Ethics, region southeast Norway, reviewed and approved the procedures for this cross-sectional study. The need for consent was waived by the ethics committee. Since our study sought to collect data relating to sexual orientation, consumption of illegal drugs and HIV diagnosis, anonymous participation was of paramount importance. When presenting to the Olafia clinic for STI health services, attendees first need to register their visit with a receptionist in a closed room within the clinic reception area. To recruit participants, the same receptionist who registered clinic attendees’ visit also screened attendees’ eligibility by informing them about the study and its eligibility criteria, and they invited eligible clinic attendees to participate in the study. Consent was provided orally and witnessed only by the receptionist in the closed room (consent was not documented in writing). Eligible clinic attendees who expressed verbal consent to participate were handed a clean paper-and-pencil questionnaire by the same receptionist, to be completed and placed in a locked box in the waiting room.

In order to ensure anonymity, and because the Olafia clinic patient records only contain information about STIs diagnosed at one of several health clinics offering STI testing and treatment in Oslo, we chose to rely on self-reported STI diagnoses over a period of one year. We used the STROBE checklist [52] in our reporting of the study.

### The survey

We piloted the first version of the survey with 20 patients who were not included in the final dataset. They provided detailed feedback on content and layout, and the final survey included 48 questions that fit on two sheets of paper. The survey was available in English and Norwegian and took 5–10 minutes to complete in the pilot. All questions, several of which were identical to questions used in the EMIS-2010 study [53], are available in a previous publication examining the full sample of both MSM and non-MSM respondents [50].

All measures were self-reported. The survey asked “In the last 12 months, how often have you taken drugs immediately preceding and/or during the sexual session (i.e., engaged in ‘chemsex’)? Drugs include crystal meth, GHB/GBL, mephedrone, ketamine, cocaine”. The five drugs were specified in accordance with existing research practices at the time [30, 54]. The survey also recorded data on sociodemographic variables (age, years lived in Norway, residence, education level, employment status, relationship status, sexual orientation, gender identity), STI health (self-reported diagnoses of chlamydia, gonorrhoea, syphilis, hepatitis C and HIV), sexual behaviours (number of sex partners, using the Internet to find sex partners, group sex participation, organised sex party participation, condom use), recency of using 13 different drugs, characteristics of chemsex engagement, mental health (including depression/anxiety, suicidal thoughts/behaviour, receiving therapy from a psychologist or psychiatrist or therapist).

We used the validated Hopkins Symptom Checklist (HCL-10) to assess anxiety and/or depression. Based on 10 symptoms that people may have experienced in the last two weeks, the checklist determines whether someone is likely to have experienced clinical depression and/or anxiety in the last two weeks. The tool has been validated for the Norwegian general population, with high reliability (Cronbach alpha = 0.88), and high validity (89% sensitivity, 98% specificity). Reliability in the current sample of MSM was 0.89. The answer options are on a 4-point Likert scale ranging from 1 “not affected” to 4 “badly affected”. An average score is calculated for individual participants who respond to at least eight of the ten symptoms by dividing the total score by the number of items, with a theoretical range of 1–4, and a reduced mental health cut-off >1.85 [55]. We asked participants identifying as gay and bisexual three different questions relating to societal homonegativity: degree of difficulty in coming as gay/bisexual, degree of outness as gay/bisexual, and internalised homonegativity. Internalised homonegativity was practically measured by asking gay and bisexual participants to rate the extent to which they regarded being homosexual or bisexual to be something intrinsically negative (independently of the prejudices of others). From the two variables measuring age and number of years lived in Norway, we created a binary variable of having lived abroad: having lived abroad never or up to a year, and having lived more than one year abroad. There is some question as to whether chemsex behaviours are picked up in larger metropolitan centres and brought back to more provincial locations such as Norway and Oslo after living abroad [56]. A myriad of differences between life in Norway and in other contexts could affect the attitudes and behaviours of MSM. An example could be international differences in social homonegativity [57].

### Data analysis

We used STATA V.16.1 (StataCorp. 2019. *Stata Statistical Software*: *Release 16*. College Station, TX: StataCorp LLC) to perform analyses. We used descriptive statistics to examine sample characteristics and statistical significance testing (Pearson’s Chi2 for binomial variables, and the Fisher’s exact test for multinomial ones) to examine differences between MSM reporting and not reporting use of either crystal meth, mephedrone, cocaine, GHB/GBL or ketamine with sex in the last 12 months. We conducted both bivariate and multiple logistic regression analyses of chemsex, including two self-reported measures of STI health (number of chlamydia/gonorrhoea/syphilis diagnoses in the last 12 months, HIV diagnosis), one mental health measure (depression/anxiety as assessed by HCL-10), and one measure of internalised homonegativity (degree to which being gay or bisexual is viewed as inherently negative). We adjusted for age, education level and having lived more than one year abroad or not. Analyses were two-tailed with significance set at the 5% level. We hypothesised there would be significant independent positive associations between chemsex engagement and self-reported number of chlamydia, gonorrhoea and syphilis-diagnoses in the last year, previous HIV diagnosis, depression/anxiety in the last two weeks, and degree of internalised homonegativity.

We set the regressions to handle missing values with listwise deletions. To minimise multicollinearity, we intentionally included variables believed to be theoretically relevant (and based on knowledge of chemsex literature) and statistically non-related. There were no concerns with model fit or positivity assumptions. The correlations among the seven independent variables were relatively low, ranging from -0.06 to 0.20.

## Results

### Description of the sample

Of the 1050 surveys handed out to male patients, 1013 (96%) were complete and could be analysed. Of these, 518 respondents were MSM, and therefore included in our analytic sample. As reported in Table 1, the mean age of the sample was 35, most had lived in Norway all their life, and nine of out ten lived in the greater Oslo area. About three quarters had some college education, 92% were employed or studying, and 59% were not in a relationship. There were minimal sociodemographic differences between men who reported chemsex engagement in the last year and men who did not, but more men who engaged in chemsex had lived abroad one year or more (44% vs. 29%). Related, of the 516 MSM responding to the question about chemsex, 17% reported sexualised use of either crystal meth, mephedrone, cocaine, GHB/GBL or ketamine in the last year. Cocaine was used by 9%, GHB/GBL by 7%, crystal meth by 4%, ketamine by 2%, and mephedrone by 2% of the respondents. Nine percent reported use of GHB/GBL, crystal meth or mephedrone in the last year. Four percent of MSM in the sample had engaged in chemsex once in the last year, 7% 2–5 times, 3% 6–10 times, 2% 11–20 times, 0.5% 21–30 times, 0% 31–40 times, 0.5% 41–50 times, 0.5% >50 times.

**Table 1:** Sociodemographic characteristics of the sample and subgroups.

	Full sample N = 518 (%)	Chemsex n = 87 (%)	No chemsex n = 429 (%)
**Age (mean [SD], range)**	34.6 (10.2), 18–79	34.6 (8.7), 21–55	34.6 (10.4), 18–79
**Lived abroad more than one year**			
No	348 (68.2)	49 (56.3)	299 (70.7)
Yes	162 (31.8)	38 (43.7)	124 (29.3)
**Current residency**			
Greater Oslo (Oslo +Akershus)	469 (91.4)	82 (95.3)	387 (90.6)
Elsewhere in Norway	31 (6.0)	2 (2.3)	29 (6.8)
Abroad	13 (2.5)	2 (2.3)	11 (2.6)
**Education**			
Primary or lower secondary	13 (2.5)	2 (2.3)	11 (2.6)
Upper secondary	94 (18.3)	20 (23.3)	74 (17.3)
Undergraduate	262 (50.6)	40 (46.5)	220 (51.4)
Postgraduate	147 (28.6)	24 (27.9)	123 (28.7)
**Employment**			
In employment	395 (76.6)	73 (83.9)	322 (75.1)
In education/student	83 (16.1)	11 (12.6)	72 (16.8)
Unemployed	22 (4.3)	3 (3.5)	19 (4.4)
Retired/disability benefits	16 (3.1)	0 (0)	16 (3.7)
**Sexual orientation**			
Homosexual	433 (83.9)	74 (85.1)	359 (83.7)
Bisexual	67 (13.0)	11 (12.6)	56 (13.1)
Heterosexual	13 (2.5)	2 (2.3)	11 (2.6)
Other	3 (0.6)	0 (0)	3 (0.7)
**Relationship status**			
In a relationship	208 (40.5)	39 (44.8)	169 (39.6)
Single	306 (59.3)	48 (55.2)	258 (60.4)

Table 2 shows respondents’ self-reported sexual behaviours, STI diagnoses, mental health, and three indicators relating to societal homonegativity. Among the full sample of MSM, in the last twelve months, 50% self-reported that they had engaged in group sex, 17% had attended an organised sex party, and 18% and 15% had been diagnosed with gonorrhoea and chlamydia infections, respectively. Nine percent self-reported an HIV diagnosis. Regarding mental health, 23% had experienced depression/anxiety in the last two weeks. With respect to societal homonegativity, half (51%) reported they had not found it difficult to come out, three quarters (79%) were open about their sexual orientation to more than half of their family members, friends, and colleagues, and 72% did not consider being gay or bisexual as something inherently negative, an indicator of internalised homonegativity. While bivariate tests for statistical significance did not suggest significant differences between MSM who engaged in chemsex and men who did not with regard to mental health or the three societal homonegativity-related variables, the two groups differed significantly in sexual behaviours, STI health (self-reported chlamydia, gonorrhoea, syphilis and HIV diagnoses) and having lived more than one year abroad (p = 0.009* in Chi2-test).

**Table 2:** Self-reported sexual behaviour and health among the sample and subgroups.

	Full sample N = 518 (%)	Chemsex n = 87 (%)	No Chemsex n = 429 (%)	P-value
**Number of sex partners** ^**a**^				
1–5	193 (37.8)	17 (19.8)	176 (41.4)	0.000*
6–10	132 (25.8)	18 (20.9)	114 (26.8)	
11–20	105 (20.6)	24 (27.9)	81 (19.1)	
21–40	58 (11.4)	21 (24.4)	37 (8.7)	
>40	23 (4.5)	6 (7.0)	17 (4.0)	
**Used condom with casual male partners** ^**a**^				
Never	30 (6.6)	7 (8.4)	23 (6.2)	0.000*
Rarely (<30%)	51 (11.3)	14 (16.9)	37 (10.0)	
Sometimes (30–70%)	69 (15.2)	23 (27.8)	46 (12.4)	
Almost always (>70%)	141 (31.1)	27 (32.5)	114 (30.8)	
Always	162 (35.8)	12 (14.5)	150 (40.5)	
**Engaged in group sex** ^**a**^				
No	255 (50.2)	13 (15.3)	242 (57.2)	0.000*
Yes	253 (49.8)	72 (84.7)	181 (42.8)	
**Attended organised sex parties** ^**a**^				
No	427 (83.6)	46 (53.5)	381 (89.7)	0.000*
Yes	84 (16.4)	40 (46.5)	44 (10.4)	
**Used the Internet to find sex partners** ^**a**^				
Never	68 (13.3)	4 (4.7)	64 (15.1)	0.041*
Rarely (<30%)	102 (20.0)	15 (17.4)	87 (20.5)	
Sometimes (30–70%)	142 (27.8)	30 (34.9)	112 (26.4)	
Almost always (>70%)	162 (31.8)	29 (33.7)	133 (31.4)	
Always	36 (7.1)	8 (9.3)	28 (6.6)	
**Sex with men only or men and women** ^**a**^				
Men only	427 (82.8)	74 (85.1)	353 (82.3)	0.532
Men and women	89 (17.3)	13 (14.9)	76 (17.7)	
**Number of chlamydia, gonorrhoea or syphilis diagnoses** ^**a**^				
0	371 (71.9)	50 (57.5)	321 (74.8)	0.000*
1	94 (18.2)	20 (23.0)	74 (17.3)	
2	36 (7.0)	8 (9.2)	28 (6.5)	
3	10 (1.9)	5 (5.8)	5 (1.2)	
4	3 (0.6)	3 (3.5)	0 (0)	
5	2 (0.4)	1 (1.2)	1 (0.2)	
**Chlamydia diagnosis** ^**a**^				
No	440 (85.3)	64 (73.6)	376 (87.7)	0.001*
Yes	76 (14.7)	23 (26.4)	53 (12.4)	
**Gonorrhoea diagnosis** ^**a**^				
No	425 (82.4)	65 (74.7)	360 (83.9)	0.040*
Yes	91 (17.6)	22 (25.3)	69 (16.1)	
**Syphilis diagnosis** ^**a**^				
No	490 (95.0)	74 (85.1)	416 (97.0)	0.000*
Yes	26 (5.0)	13 (14.9)	13 (3.0)	
**Hepatitis C diagnosis** ^**b**^				
No	492 (97.4)	83 (98.8)	409 (97.1)	0.380
Yes	13 (2.6)	1 (1.2)	12 (2.9)	
**HIV diagnosis** ^**b**^				
No	453 (91.5)	66 (78.6)	387 (94.2)	0.000*
Yes	42 (8.5)	18 (21.4)	24 (5.8)	
**Depression/anxiety** ^**c**^				
No	364 (77.0)	62 (77.5)	302 (76.8)	0.899
Yes	109 (23.0)	18 (22.5)	91 (23.2)	
**Currently receiving therapy from a psychologist/ psychiatrist/ therapist**				
No	408 (89.3)	71 (91.0)	337 (88.9)	0.584
Yes	49 (10.7)	7 (9.0)	42 (11.1)	
**Seriously considered suicide** ^**a**^				
No	467 (95.5)	79 (95.2)	388 (95.6)	0.877
Yes	22 (4.5)	4 (4.8)	18 (4.4)	
**Coming out as gay or bisexual—perceived degree of difficulty**				
Too difficult: not out	35 (7.8)	4 (5.3)	31 (8.5)	0.521
Difficult	183 (41.4)	29 (38.2)	154 (42.1)	
Neither easy nor difficult	157 (35.5)	28 (36.8)	129 (35.3)	
Easy	67 (15.2)	15 (19.7)	52 (14.2)	
**Degree to which sexuality is disclosed to family, friends and colleagues/fellow students (outness)**				
Nobody knows	14 (3.1)	2 (2.6)	12 (3.2)	0.989
Few know	46 (10.2)	7 (9.1)	39 (10.4)	
Less than half know	37 (8.2)	7 (9.1)	30 (8.0)	
More than half know	40 (8.8)	6 (7.8)	34 (9.0)	
All or almost all know	316 (69.8)	55 (71.4)	261 (69.4)	
**Degree to which being gay or bisexual is inherently negative (internalised homonegativity)**				
Very negative	12 (2.6)	2 (2.6)	10 (2.7)	0.499
Somewhat negative	116 (25.6)	16 (20.8)	100 (26.5)	
Not sure if negative or not	10 (2.2)	3 (3.9)	7 (1.9)	
Not negative at all	316 (69.6)	56 (72.7)	260 (69.0)	

^a^In the last year

^b^Ever

^c^In the last two weeks

*p<0.05 in Pearson’s Chi2-test for binomial variables, or in the Fisher’s exact test for multinomial variables.

### Associations between chemsex, STI health, mental health and internalised homonegativity

Table 2 highlights consistent differences between MSM who engage in chemsex and MSM who do not, with respect to sexual behaviour and STI diagnoses. The proportions reporting any number of chlamydia, gonorrhoea or syphilis diagnoses in the last year are consistently higher among those engaging in chemsex. Similarly, 21% of MSM participants engaging in chemsex in the last year reported an HIV diagnosis, in contrast to 6% of those who did not engage in chemsex. As seen in Table 3, our bivariate logistic regression analyses produced significant positive associations between chemsex and the number of self-reported chlamydia, gonorrhoea or syphilis diagnoses in the last year (odds ratio [OR] = 1.70, 95%CI = 1.33–2.18) and HIV diagnosis (OR = 4.40, 95%CI = 2.26–8.55), but not for the two variables concerning mental health and internalised homonegativity. In both the bivariate and multivariate regression analyses, there was a significant positive association between chemsex and having lived more than one year abroad (adjusted OR [aOR] = 2.10, 95%CI = 1.20–3.65). In the multivariate logistic regression analysis, number of self-reported chlamydia/gonorrhoea/syphilis diagnoses (aOR = 1.63, 95%CI = 1.21–2.18) and HIV diagnosis (aOR = 3.26, 95%CI = 1.37–7.76) remained significantly associated with chemsex, after having adjusted for age, education level, and having lived more than one year abroad.

**Table 3:** Logistic regressions of chemsex and self-reported STI health, mental health and internalised homonegativity.

	Univariate		Multivariate	
Independent variables	OR (95% CI)	P-value	aOR (95% CI)	P-value
Number of chlamydia / gonorrhoea / syphilis diagnoses^a^	1.70 (1.33–2.18)	0.000*	1.63 (1.21–2.18)	0.001*
HIV diagnosis^b^	4.40 (2.26–8.55)	0.000*	3.26 (1.37–7.76)	0.007*
Depression /anxiety^c^	0.96 (0.54–1.71)	0.899	1.05 (0.55–2.01)	0.878
Internalised homonegativity	1.12 (0.85–1.46)	0.420	1.23 (0.90–1.67)	0.190
Age	1.00 (0.98–1.02)	0.993	0.98 (0.96–1.02)	0.324
Education	0.90 (0.66–1.21)	0.479	0.95 (0.66–1.37)	0.800
Lived >1 year abroad	1.87 (1.17–3.00)	0.009*	2.10 (1.20–3.65)	0.009*

Analytic n = 407 in multivariate model due to listwise deletion.

^a^in the last year

^b^ever

^c^in the last two weeks

*statistically significant regression result (p<0.05)

## Discussion

In our cross-sectional study among 518 MSM recruited from a free hospital walk-in STI clinic in Oslo, 17% reported sexualised use of either crystal meth, mephedrone, cocaine, GHB/GBL or ketamine during the last year. Our results show that chemsex engagement is relatively common among MSM in Oslo, that the most frequently used drugs with sex are cocaine and GHB/GBL, and that chemsex is more common among participants living with HIV, those reporting higher frequencies of chlamydia/gonorrhoea/syphilis diagnoses, and those having lived more than one year abroad. There was no significant association between chemsex engagement and depression/anxiety or internalised homonegativity.

It is difficult to compare our results with similar chemsex studies among MSM, due to the lack of probabilistic sampling, the variability in operationalisations of chemsex, in recruitment of MSM study participants, and in measurement of variables such as depression. Additionally, Nordic countries are unique in their consistently high ranking in life satisfaction [58–60], and the social democratic model for governing and organising their societies [61]. Valid comparisons with results of similar research in other countries or geographical regions are therefore difficult to make. Our study does, however, usefully expand the research on chemsex among MSM beyond the usual countries of the US, the UK, the Netherlands, and Australia [15], and it offers important considerations for practice and research.

According to recent reviews of the literature on chemsex from high income countries, prevalence estimates in the last 12 months range from 3% to 29% [12], and in the UK from 4% to 41% [62]. Among MSM attending STI testing clinics, chemsex engagement has been reported at 17–27% [12]. In a UK study from an STI clinic that defined chemsex as we did, 17% of study participants reported chemsex engagement in the last 7 months [30]. In a study among MSM attending an STI clinic in Ireland, the proportion reporting GHB/GBL use with sex in the last year was more than twice as high as in our study (15% vs 7%) [8]. Similar higher rates were found also for cocaine, crystal meth, ketamine, and mephedrone. Lastly, in a study from an STI clinic at the Public Health Service of the Netherlands, 25% of MSM participants had used crystal meth, mephedrone or GHB/GBL with sex in the last six months [24], which is considerably higher than the 9% reported by our sample of MSM in a year. Thus, in these UK, Irish and Dutch studies, which are comparable to our study, chemsex engagement and use of specific drugs with sex are consistently higher than the rates we found. Results from a large Internet-based survey corroborate the comparably lower use of chemsex drugs among MSM in Oslo, however not measured specifically in relation to use with sex [63]. These results suggest that chemsex may be less common among MSM residing in Norway than in other regions of Europe. One relevant factor when evaluating differences across geographical locations is the types of drugs available in the different drug markets [3, 12].

The higher rate of chemsex engagement in our sample compared to the 7% rate reported by a more generally selected Internet population of MSM residing in Norway [25, 26] is likely attributable to differences in study populations and operationalisations of chemsex. The discrepancy is likely affected by the tendency among those engaging in chemsex to generally experience higher rates of bacterial STI diagnoses (such as chlamydia, gonorrhoea and syphilis), evidenced across a range of MSM populations [7, 14, 30–35]. In turn, they attend STI clinics in disproportionately greater proportions than the general MSM population. Related, we found that MSM in our sample reporting chemsex engagement were more likely to report more sex partners, group sex engagement and organised sex party attendance in the last 12 months, and less likely to report consistent condom use. These sample variations in sexual behaviour between MSM engaging and not engaging in chemsex are consistent with findings of MSM attending STI health clinics [12]. In the view of some researchers, chemsex almost by definition involves group sex, sex party attendance and a high number of sex partners (Reviewer), suggesting that chemsex may become increasingly relevant to MSM health as pre-exposure prophylaxis (PrEP), a medicalised form of HIV prevention, is becoming more easily available to an increasing number of MSM across the world. Data from our own survey illustrates this relevance, as 77% of those engaging in chemsex and not reporting an HIV diagnosis expressed an interest in PrEP if it were to be made available through the public health system, and 56% of those who had not engaged in chemsex nor reported an HIV diagnosis expressed the same interest. Furthermore, we found that roughly half of those we classified as having engaged in chemsex had used neither crystal meth, mephedrone nor GHB/GBL with sex. This highlights the importance of including or not including drugs such as cocaine or ecstasy/MDMA in study definitions of chemsex. A growing number of researchers is highlighting this methodological issue in the MSM chemsex literature field [7, 16, 23, 24, 64].

Another important finding in our study was the strong association between chemsex and HIV diagnosis, as MSM reporting an HIV diagnosis were found to have 3.3 times higher odds of chemsex participation. The result confirms a consistent finding in primary studies from STI clinic settings in other countries [7, 14, 30–33, 35]. It also mirrors an older Internet-based study among MSM in Norway from 2010, which found that MSM self-reporting previous HIV diagnosis were more likely to engage in sex while being under the influence of marihuana, prescription drugs, ecstasy, LSD, GHB, cocaine, heroin, amphetamines or crystal meth [65]. Albeit the association was somewhat weaker, a related finding of our study was the significant association between chemsex participation and self-reported number of chlamydia, gonorrhoea or syphilis diagnoses, which also confirms results from similar studies [14, 31]. While the direction of the relationship between chemsex and HIV diagnosis cannot be established as of yet, there is some merit to presuming that bacterial STIs (such as chlamydia, gonorrhoea and syphilis) are unlikely to cause chemsex engagement on a theoretical basis [66]. Irrespective of directionality, given the higher occurrence of HIV, chlamydia, gonorrhoea and syphilis among MSM who engage in chemsex, support and treatment options for MSM who practice chemsex, and perceive a need for support in managing or reducing their engagement, must be made available. As a first step, healthcare professionals who serve MSM need to be informed about chemsex. Further, addressing sexualised drug use must be part of clinical, public health and outreach activity, ideally incorporating harm reduction strategies. There is some evidence that the majority of STI clinic clients are comfortable addressing substance use with an STI clinic provider [67]. There are positive experiences with harm reduction strategies [68], treatment approaches integrating MSM peer-support, and professional support [69]. In Australia, promising findings for community-led approaches to chemsex that combine support services, health promotion activities, peer education and policy work exist [70].

While we found support for our hypotheses of a positive association between chemsex and previous HIV diagnosis and number of chlamydia/gonorrhoea/syphilis diagnoses, our multivariate analysis did not support our hypotheses of a positive association with depression/anxiety and internalised homonegativity. In contrast to the vast number of studies on STIs among MSM who engage in chemsex, research on mental health and measures of homonegativity is scarce [13–15]. Furthermore, quantitative studies show mixed results [13, 14]. We cannot rule out potential associations between chemsex and reduced mental health, or between chemsex and homonegativity, however, and encourage further research on both as potential drivers and/or consequences of chemsex. We agree with Tomkins and colleagues [14], who suggest that chemsex may be a tool to alleviate pre-existing mental and sexological health functioning problems, and that not only quantitative, but also qualitative evidence would be necessary to determine the complex interrelations between these different factors. We encourage future research to disentangle which factors are drivers, consequences and/or confounders, and under which circumstances. Considering that chemsex has become a part of the sexual lives of many MSM in different countries, including Norway, we believe there is not one, but multiple chemsex practices among MSM that involve different sets of health risks and benefits, echoing Santoro and colleagues [23]. It is crucial to examine these issues further. Lastly, we found that chemsex was significantly associated with having lived at least one year abroad. This indicates that previous exposure to cultures other than the local culture of the study location(s) could be relevant for understanding chemsex engagement. It is possible, as other researchers have suggested [56], that chemsex behaviours are ‘learnt’ when living in larger metropolitan cities and continued upon return to more provincial locations such as Norway and Oslo. This would be an interesting topic in future research.

Our research comes with some limitations. The cross-sectional study design does not allow for any inferences regarding causality, and it is possible that our findings are limited by measurement bias, especially with respect to the variable internalised homonegativity, which was chosen for reasons of brevity. Using an instrument such as the 7-item Internalised Homonegative Scale would have been better [71, 72]. Our reliance on self-report may limit our findings further, including measurement bias. Recruitment was through a walk-in STI clinic in Oslo. Our results may not be generalizable to MSM not seeking STI health services, living in rural areas, who have lived much of their lives or currently live outside Norway, nor MSM who left school relatively early. It is also worth noting that after the survey was carried out, PrEP has become freely available in Norway. Although our use of multivariate analyses is a study strength, our population estimates would be more precise with more study participants, or a higher participant proportion reporting chemsex within a sample of the same size. We believe separate regression analyses for each individual drug is a critical next step in research on chemsex among MSM, but were unable to do so because of the relatively low sample share reporting use of one specific drug with sex. As indicated in a few studies [23, 38], there may be important health differences between sexualised use of drugs such as crystal meth, cocaine and GHB/GBL. Future studies should also consider conducting latent class analyses based on sexualised use of individual drugs. According to Lafortune and colleagues [13], such analyses may reveal multiple profiles of sexual and non-sexual substance use among MSM, as well as their interplay with other variables.

## Conclusion

In our study of crystal meth, mephedrone, cocaine, GHB/GBL or ketamine use with sex among MSM attending an STI clinic in Oslo, findings indicate that health care providers need to be aware of chemsex engagement, and the link with both HIV diagnosis and frequency of chlamydia/gonorrhoea/syphilis diagnoses among MSM. Special care should be afforded to MSM who disclose they use drugs with sex, in order to facilitate access to services that could help address any problematic aspects of the behaviour and potential underlying problems. Among MSM living with HIV in particular, specific patterns of sexualised drug use may represent a behavioural marker of problematic sexological health, and should be examined in future studies.
